# Prolonged maternal exposure to glucocorticoids alters selenoprotein expression in the developing brain

**DOI:** 10.3389/fnmol.2023.1115993

**Published:** 2023-03-24

**Authors:** Pamela Toh, Lucia A. Seale, Marla J. Berry, Daniel J. Torres

**Affiliations:** Pacific Biosciences Research Center, School of Ocean and Earth Science and Technology, University of Hawaii at Manoa, Honolulu, HI, United States

**Keywords:** corticosterone, glucocorticoid, neurodevelopment, selenium, selenoprotein

## Abstract

Aberrant activation of the stress-response system in early life can alter neurodevelopment and cause long-term neurological changes. Activation of the hypothalamic–pituitary–adrenal axis releases glucocorticoids into the bloodstream, to help the organism adapt to the stressful stimulus. Elevated glucocorticoid levels can promote the accumulation of reactive oxygen species, and the brain is highly susceptible to oxidative stress. The essential trace element selenium is obtained through diet, is used to synthesize antioxidant selenoproteins, and can mitigate glucocorticoid-mediated oxidative damage. Glucocorticoids can impair antioxidant enzymes in the brain, and could potentially influence selenoprotein expression. We hypothesized that exposure to high levels of glucocorticoids would disrupt selenoprotein expression in the developing brain. C57 wild-type dams of recently birthed litters were fed either a moderate (0.25 ppm) or high (1 ppm) selenium diet and administered corticosterone (75 μg/ml) *via* drinking water during postnatal days 1 to 15, after which the brains of the offspring were collected for western blot analysis. Glutathione peroxidase 1 and 4 levels were increased by maternal corticosterone exposure within the prefrontal cortex, hippocampus, and hypothalamus of offspring. Additionally, levels of the glucocorticoid receptor were decreased in the hippocampus and selenoprotein W was elevated in the hypothalamus by corticosterone. Maternal consumption of a high selenium diet independently decreased glucocorticoid receptor levels in the hippocampus of offspring of both sexes, as well as in the prefrontal cortex of female offspring. This study demonstrates that early life exposure to excess glucocorticoid levels can alter selenoprotein levels in the developing brain.

## 1. Introduction

The hypothalamic–pituitary–adrenal (HPA) axis is responsible for the induction of allostatic processes throughout the body. In response to stressors, the HPA axis activates a biochemical cascade that stimulates the adrenal glands to secrete glucocorticoids (GC). These steroid hormones–primarily cortisol in humans, and corticosterone (CORT) in rodents–go on to regulate a host of systems involved in the stress response (Sapolsky et al., 2000). While basal levels of GCs are necessary for maintaining normal homeostasis (Herman et al., 2016), chronically high levels of circulating GCs have been associated with disrupted energy production (Sapolsky, 1986) and increased disease susceptibility (Conrad, 2008; Shimba and Ikuta, 2020; Grilo et al., 2021). Elevated GC levels in early-life can be particularly harmful, resulting in lasting effects on cognitive function and emotional regulation (Lupien et al., 2009).

GCs are known to cause cellular damage through multiple mechanisms, one of which involves the dysregulation of mitochondrial activity and the subsequent accumulation of reactive oxygen species (ROS) (Cadenas and Davies, 2000; Du et al., 2009; Morella et al., 2022). The brain is particularly susceptible to oxidative stress (Costantini et al., 2011) and utilizes selenoproteins as a major form of antioxidant defense. Among the most well-studied selenoproteins are several members of the glutathione peroxidase (GPX) sub-family, which is responsible for reducing peroxide species. Selenoproteins incorporate the essential micronutrient, selenium (Se), in the form of a highly reactive selenocysteine residue. The relatively low pKa (~5.2) of this non-standard amino acid allows for selenoproteins to act as particularly effective antioxidant enzymes (Arner, 2010). Due to their role in redox regulation, Se and selenoproteins are critical to brain function and development (Solovyev, 2015; Nicholson et al., 2022; Schweizer and Fabiano, 2022), and expression levels have been shown to be altered by GCs (Mcintosh et al., 1998a; Torres et al., 2021).

Se is critical during neurodevelopment, and Se availability during gestation and early-life is largely dependent on the dietary intake of the mother (Tsuji et al., 2021; Schweizer and Fabiano, 2022). The recommended daily allowance of Se is raised during pregnancy (Monsen, 2000) as maternal Se and GPX activity decrease in response to the redistribution of nutrients and resource demands of a developing fetus (Zachara et al., 1986; Mihailovic et al., 2000; Pieczynska and Grajeta, 2015; Ambroziak et al., 2017; Schomburg, 2021). Lactation also imposes a persistent nutritional demand on maternal Se that has the potential to maintain maternal Se deficiency into postpartum (Varsi et al., 2017). Indeed, both pregnant and nursing mothers, as well as infants, are at risk for Se deficiency, and low maternal Se during pregnancy has been associated with neurodevelopmental deficits in offspring (Varsi et al., 2017). Given the interplay between Se and the energetically demanding landscape of pregnancy and early development, stressors during this vulnerable period may trigger unique biological responses. Given that past literature suggests that GCs can alter selenoprotein expression, this interaction could contribute to the negative impact of elevated GC levels during development. We sought to determine whether the intersection between high GCs and selenoprotein expression occurs in pregnancy by administering CORT to nursing mouse dams and measuring selenoprotein levels in the brains of the offspring.

## 2. Materials and methods

### 2.1. Animal care and experimental design

**Figure 1:**
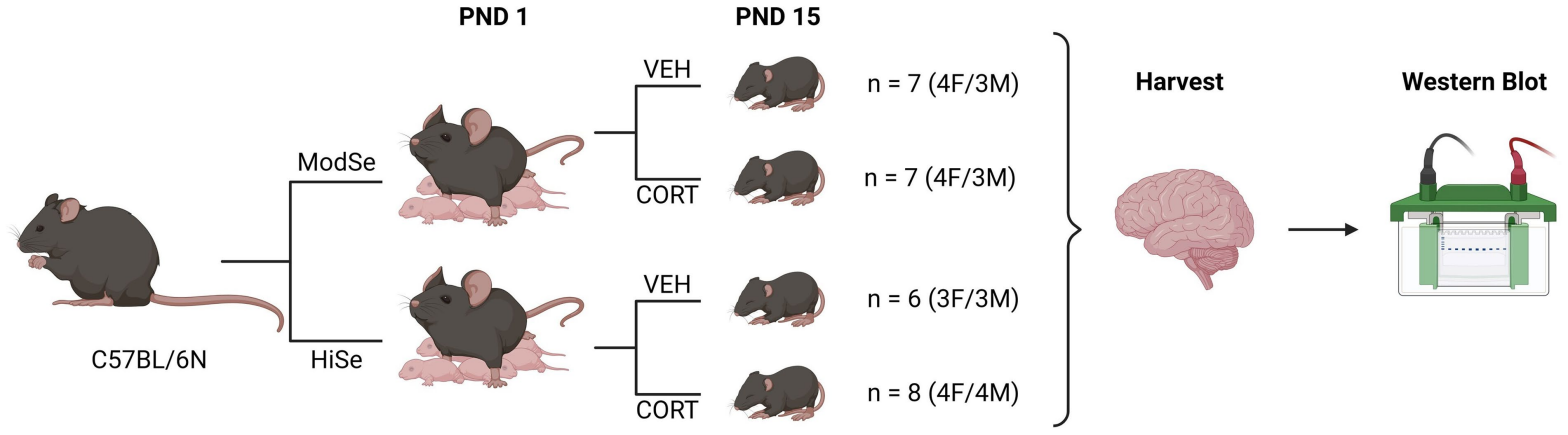
Summary of experimental design. Wild-type C57BL/6 N mice were mated while being fed chow that contained either a moderate selenium (ModSe, 0.25 ppm) or high selenium (HiSe, 1 ppm) content. Following the birth of a litter, on postnatal day 1 (PND1), either corticosterone (CORT, 75 μg/ml), or vehicle control (1% grain alcohol) were added to the drinking water, and replaced twice weekly. On PND15, the entire litter was sacrificed and brain regions were harvested for western blot analysis.

Experiments were approved by the UH Manoa Institutional Animal Care and Use Committee (protocol number 09–871) in compliance with the ARRIVE guidelines and the National Research Council’s *Guide for the Care and Use of Laboratory Animals*. Wild-type (WT) C57/BL6N mice (RRID:IMSR_JAX:005304) were maintained on a 12/12 h on/off light cycle with *ad libitum* access to food and water. Mice were fed diets containing either 0.25 ppm (“Moderate selenium”; Envigo, Cat# TD.120504) or 1 ppm sodium selenite (“High selenium,” Envigo, Cat# TD.03249). Nursing dams were given drinking water containing either corticosterone (75 μg/ml) or vehicle control (1% grain alcohol) from postnatal days (PND) 1–15. This method results in excess CORT being delivered to postnatal pups, and *via* maternal milk (Angelucci et al., 1985). Selenium is also supplied to the offspring through the milk (Hill et al., 2014). Brain tissues were harvested from male and female offspring following CO_2_ asphyxiation on PND 15 and flash-frozen in liquid nitrogen for protein analysis. Protein levels were quantified for the glucocorticoid receptor (GCR), selenoprotein W (SELENOW), GPX1, and GPX4 in the prefrontal cortex, hypothalamus, and hippocampus. The experimental design is summarized in Figure 1.

### 2.2. Gel electrophoresis and Western blotting

Dissected brain parts were pulverized on dry ice and protein was extracted from the homogenized powder using CelLytic MT Mammalian Tissue Lysis Buffer (Sigma, Cat# C3228) containing 1:100 protease inhibitor cocktail (Cell Signaling, Cat# 5872). Following sonication, samples were centrifuged and the supernatant collected for western blot analysis. 80 μg of protein were separated *via* electrophoresis on 4–20% gradient polyacrylamide TGX gels (BIO-RAD, Cat# 5671094), and then transferred to Immobilon-FL polyvinylidene difluoride membrane with 0.45 μm pore size (Millipore, Cat# IPFL00010). Membranes incubated in Intercept (PBS) Blocking Buffer (LI-COR, Cat# 927–70,001), after which target proteins were detected using primary antibodies and infrared fluorescent secondary antibodies. The Odyssey XF Imaging System (LI-COR) was used to image the blots and data were analyzed using Image Studio software (LI-COR, RRID:SCR_015795).

The following primary antibodies were used: anti-Glucocorticoid receptor monoclonal antibody [BuGR2] (1:1,000; ThermoFisher Scientific, Cat# MA1-510, RRID:AB_325427), anti-Glutathione peroxidase 1 polyclonal antibody (1:2,000; R&D Systems, Cat# AF3798, RRID:AB_2112108), anti-Glutathione peroxidase 4 monoclonal antibody [EPNCIR144] (Epitomics, Cat# ab125066, RRID:AB_10973901), anti-Selenoprotein W polyclonal antibody (1:1,000; Rockland, Cat# 600-401-A29, RRID:AB_2285666), and anti-β-actin monoclonal antibody [8H10D10] (1:5,000; Cell Signaling, Cat# 3700, RRID:AB_2242334). Secondary antibodies used were highly cross-adsorbed IRDye conjugated secondary antibodies from LI-COR: 800CW donkey anti-goat (1:10,000; Cat# 926–32,214, RRID:AB_621846), 800CW donkey anti-mouse (1:10,000; Cat# 926–32,212, RRID:AB_621847), 680LT donkey anti-mouse (1,10,000; Cat# 926–68,022, RRID:AB_10715072), and 680LT donkey anti-rabbit (1,10,000; Cat# 926–68,023, RRID:AB_10706167).

### 2.3. Statistical analysis

Western blot data were normalized to the control condition, moderate selenium-fed, vehicle treated, and displayed as a percent change from baseline. Data were analyzed using 2-way ANOVA, followed by Tukey’s multiple comparisons post-hoc, to test for effects of both selenium diet and corticosterone treatment. Comparisons were made within individual brain regions and within a single sex. The following criteria were used for significance: at *p* < 0.05 (*), *p* < 0.01 (**), *p* < 0.001 (***), and *p <* 0.0001 (****). All statistical analysis was performed using GraphPad Prism 8 software (RRID:SCR_002798). Sample sizes were determined by the number of neonates in each litter. A single litter was used for each experimental group.

## 3. Results

**Figure 2:**
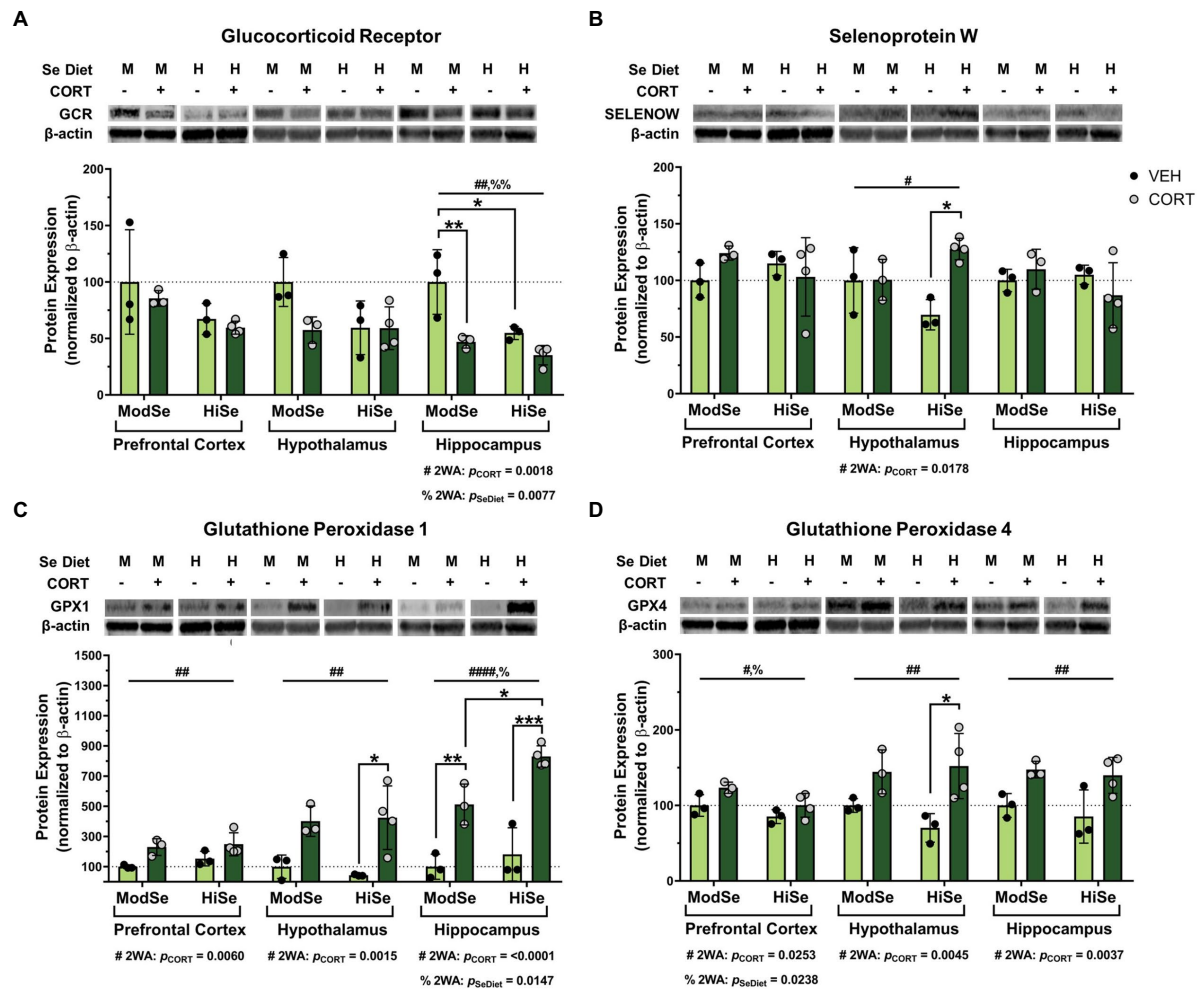
Western blot analysis of the prefrontal cortex (PFC), hypothalamus (HYP), and hippocampus (HPC) of male mice on either a moderate (ModSe) or high selenium (HiSe) diet being treated with either CORT or vehicle control (VEH). **(A)** Percentage change in glucocorticoid receptor (GCR) protein levels. HPC: CORT and HiSe consumption both independently decreased GCR levels [p_CORT_ = 0.0018, *F*_(1,9)_ = 15.15. p_SeDiet_ = 0.0077, *F*_(1,9)_ = 11.68]. HiSe diet without CORT decreased GCR to the level of CORT-exposed ModSe mice, and GCR expression in both groups was significantly lower than the ModSe/VEH group **(B)** Percentage change in SELENOW protein levels. HYP: CORT increased SELENOW levels [p_CORT_ = 0.0178, *F*_(1,9)_ = 8.378], particularly within HiSe offspring. **(C)** Percentage change in GPX1 protein levels. CORT significantly increased GPX1 levels in the PFC [p_CORT_ = 0.0060, *F*_(1,9)_ = 12.79], HYP [p_CORT_ = 0.0015, *F*_(1,9)_ = 20.28] and HPC [p_CORT_ = <0.0001, *F*_(1,9)_ = 63.59]. HiSe diet consumption increased GPX1 in the HPC: p_SeDiet_ = 0.0147, *F*_(1,9)_ = 9.052. GPX1 reached the highest levels within the HiSe/CORT condition as this group was significantly higher than the ModSe/CORT group. **(D)** Percentage change in GPX4 protein levels. GPX4 was elevated in response to CORT in all brain regions: PFC [p_CORT_ = 0.0238, *F*_(1,9)_ = 7.372], HYP [p_CORT_ = 0.0045, *F*_(1,9)_ = 14.08], and HPC [p_CORT_ = 0.0037, *F*_(1,9)_ = 15.15]. Unexpectedly, maternal Se intake negatively impacted GPX4 levels in the PFC: p_SeDiet_ = 0.0253, *F*_(1,9)_ = 7.171. Individual data points are shown and bars indicate the mean ± standard error of the mean. All comparisons were made within each brain region for each protein *via* 2-way ANOVA, where # indicates a significant effect of CORT and % a significant effect of Se diet, with Tukey’s multiple comparisons used as a post-hoc test: *p < 0.05, **p < 0.01, ***p < 0.001. *N* = 3, 3, 3, 4 indicated in graph.

This study tested the hypothesis that GC consumption by lactating dams can alter selenoprotein expression in the brains of offspring. Lactating dams consumed CORT in the drinking water beginning at PND 1 and the brains of the pups were dissected at PND 15. Western blot analysis revealed that maternal CORT consumption significantly decreased GCR protein levels in the hippocampus of male offspring (Figure 2A). Interestingly, maternal consumption of high selenium chow had an independent negative effect on GCR expression in the hippocampus. The levels of SELENOW were increased by CORT in the male offspring hypothalamus, with the biggest difference observed within the offspring44 of high Se-fed mothers (Figure 2B). The administration of CORT had the most profound effects on the expression of the GPXs. Protein levels of GPX1 were higher in all three brain regions, the prefrontal cortex, hypothalamus, and hippocampus, of male offspring of CORT-exposed mothers, compared to their vehicle control counterparts (Figure 2C). Maternal high Se consumption significantly increased GPX1 in the hippocampus compared to moderate Se consumption, but not in the prefrontal cortex or hypothalamus. GPX4 levels were also significantly elevated by CORT in all brain regions measured (Figure 2D). Unexpectedly, GPX4 was found to be decreased by high Se in the prefrontal cortex of male offspring.

**Figure 3:**
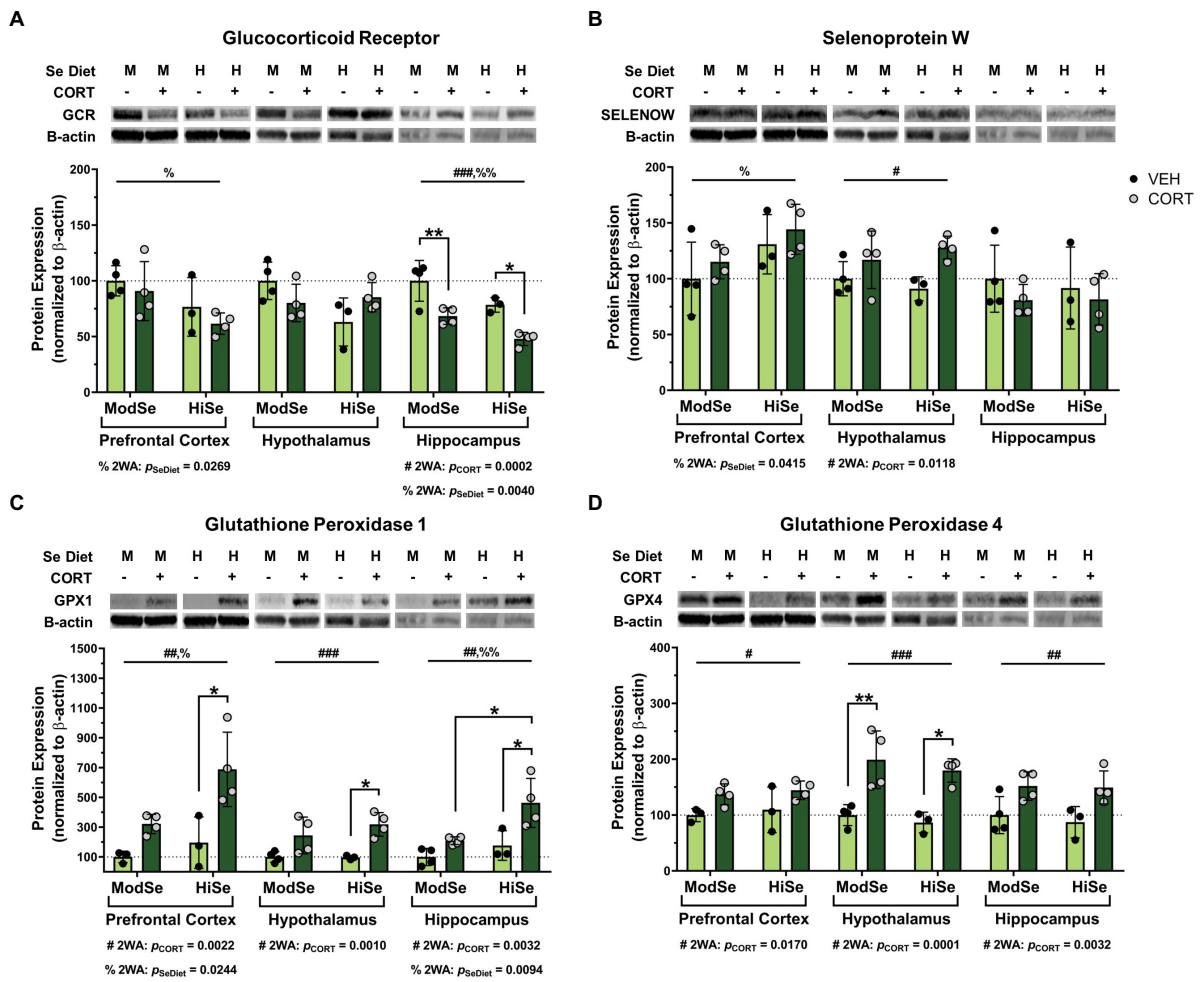
Western blot analysis in the prefrontal cortex (PFC), hypothalamus (HYP), and hippocampus (HPC) of female mice on a moderate (ModSe) or high selenium (HiSe) diet being treated with either CORT or vehicle control (VEH). **(A)** Percentage change in glucocorticoid receptor (GCR) protein levels. CORT significantly reduced GCR in the HPC [p_CORT_ = 0.0002, *F*_(1,11)_ = 28.70]. As with male offspring, maternal consumption of HiSe decreased GCR levels in the HPC [p_SeDiet_ = 0.0040, *F*_(1,11)_ = 13.14], however this effect was also observed in the PFC[p_SeDiet_ = 0.0269, *F*_(1,11)_ = 6.514]. **(B)** Percentage change in SELENOW protein levels. CORT significantly increased SELENOW levels in the HYP: p_CORT_ = 0.0118, *F*_(1,11)_ = 9.075. HiSe also increased SELENOW in the PFC: p_SeDiet_ = 0.0415, *F*_(1,11)_ = 5.322. **(C)** Percentage change in GPX1 protein levels. CORT increased GPX1 levels in all three brain regions: PFC [p_CORT_ = 0.0022, *F*_(1,10)_ = 16.68], HYP [p_CORT_ = 0.0010, *F*_(1,11)_ = 19.78], and HPC [p_CORT_ = 0.0032, *F*_(1,11)_ = 14.02]. HiSe increased GPX1 in both the PFC [p_SeDiet_ = 0.0244, *F*_(1,10)_ = 7.009] and the HPC [p_SeDiet_ = 0.0094, *F*_(1,11)_ = 9.852]. **(D)** Percentage change in GPX4 protein levels. CORT elevated GPX4 levels in all brain regions: PFC[p_CORT_ = 0.0170, *F*_(1,10)_ = 8.175], HYP[p_CORT_ = 0.0001, *F*_(1,11)_ = 34.09], and HPC[p_CORT_ = 0.0032, *F*_(1,11)_ = 14.03]. All comparisons were made within each brain region for each protein *via* 2-way ANOVA, where # indicates a significant effect of CORT and % a significant effect of Se diet, with Tukey’s multiple comparisons used as a post-hoc test: *p < 0.05, **p < 0.01, ***p < 0.001. *N* = 4, 4, 3, 4 indicated in graph.

Overall, the brains of female offspring were affected similarly to males, with some noted differences. GCR levels were decreased by both CORT and high Se in the hippocampus of female offspring (Figure 3A). The reducing effect of high maternal Se consumption on GCR levels was also observed in the female prefrontal cortex. SELENOW levels were augmented by CORT in the hypothalamus, as well as by high Se in the prefrontal cortex (Figure 3B). Administration of CORT increased the levels of both GPX1 and GPX4 in all brain regions of female offspring (Figures 3C,D). High Se only impacted GPX1 levels, however, in both the prefrontal cortex and hippocampus.

## 4. Discussion

The observations described herein demonstrate that the consumption of exogenous CORT by nursing dams can influence the neurobiology of the offspring, including by altering the GCR protein content of three glucocorticoid-sensitive brain regions. Chronic overactivation of the HPA axis is known to have a dampening effect on the negative feedback loop that limits glucocorticoid secretion (Conrad, 2008; Godoy et al., 2018). This regulatory loop involves brain-resident GCR and in the present study, maternal CORT exposure caused a downward trend in brain GCR levels that was statistically significant in the hippocampus of offspring of both sexes. This finding is consistent with past literature on the negative regulatory effect of GCs on hippocampal GCR (Conrad, 2008). This phenomenon is hypothesized to be a protective adaptation as GCs can increase the risk of glutamate excitotoxicity in the hippocampus (Stein-Behrens et al., 1994; Treccani et al., 2014). Overall, these results reflect the vulnerability of the hippocampus, which contains the highest density of GCR expression at baseline amongst all major regions of the rodent brain (Mcewen et al., 1968; Mcewen, 2007). Although GCR expression trended downward in the hypothalamus, the command center of the HPA axis, the data did not reach statistical significance. This outcome is congruent with previous work that found that CORT administered to adult mice *via* drinking water for 21 days markedly decreased hippocampal GCR levels while causing a statistically non-significant downward trend in the hypothalamus (Angoa-Perez et al., 2021). Thus, our findings in regard to GCR expression in the brains of offspring of CORT-exposed dams are consistent with the observed effects of excess GCs on the adult rodent brain.

Although the maternal consumption of a high selenium diet did not modify the influence of CORT on brain GCRs in the offspring, it did have an independent effect on GCR levels, lowering them in the hippocampus of both sexes and the prefrontal cortex of females. Multiple groups have reported on the ability of selenium to “normalize” the HPA axis in rodents under stress conditions, including preventing the downregulation of GCR in the brain (Torres et al., 2021). In one of these studies, performed on mice undergoing acute-restraint stress, selenium by itself caused an increase in GCR levels in the prefrontal cortex and a non-significant upward trend in the hippocampus of non-stressed control animals, in juxtaposition with our data (Casaril et al., 2019). This study utilized a synthetic organo-compound that contains selenium, however, and although it has known antioxidant properties (Casaril et al., 2017), the selenium residue is likely not metabolized and utilized for selenoprotein expression in the same manner as the naturally-occurring selenium contained within food. Moreover, the GCR upregulation was an acute result of a singular injection of the seleno-compound, as opposed to the long-term result of dietary selenium intake, as in our study.

A review of the literature produces few reports of the effects of selenium on the GCR. One study found that the consumption of high amounts of selenomethionine reduced GCR gene expression in the livers of white sturgeon fish (Patterson et al., 2017). Although the concentration of selenomethionine used is thought to be toxic, GCR transcripts also trended downward, albeit non-significantly, in response to lower selenomethionine doses. The authors hypothesized that the decrease in GCR mRNA may have been an adaptation to elevated GCR protein levels, but did not measure the amount of protein present. Another report described the ability of selenite to directly inhibit the ligand-binding ability of the rat liver GCR *in vitro* (Tashima et al., 1989). To our knowledge, ours is the first report of an effect of dietary selenium consumption on GCR levels in the brain. Multiple lines of evidence suggest that the functionality of the GCR is affected by redox balance on numerous levels (Tanaka et al., 1999), as oxidative conditions seem to inhibit GCR activity (Okamoto et al., 1998, 1999). One potential explanation for the decrease in brain GCR in the offspring of high selenium-fed dams may be that a greater availability of selenium creates a redox environment that promotes GCR functionality. Perhaps GCR protein levels are consequently reduced as an adaptation to prevent cellular over-sensitization to GCs. Further investigation into the influence of dietary selenium on the GCR is warranted as our findings indicate that selenium status may alter the response of the developing brain to GCs.

Maternal consumption of CORT caused a robust augmentation of GPX1 expression in all three brain regions. Through its reduction of hydrogen peroxide, GPX1 plays a central role in preventing oxidative stress (Handy and Loscalzo, 2022). Considering that elevated GCs can make neurons more vulnerable to oxidative damage by raising basal reactive oxygen species (ROS) levels (Mcintosh and Sapolsky, 1996; Behl et al., 1997; Spiers et al., 2014), the increase in GPX1 may be a protective adaptation. Glucocorticoids are known to impair antioxidant enzymes throughout the body (Dougall and Nick, 1991; Asayama et al., 1992; Kratschmar et al., 2012; An et al., 2016), including in the brain (Mcintosh et al., 1998a,b). Past studies have produced contrasting data on the effects of GCs on GPX1. Acute (up to 24 h) GC application was shown to decrease GPX1 transcript and activity levels in cultured rat hippocampal cells (Patel et al., 2002; You et al., 2009), and two weeks of *in vivo* CORT injections reduced hippocampal GPX1 activity in rats (Sato et al., 2010). Additionally, while immobilization stress decreased GPX1 activity, cold-induced stress elevated GPX1 activity in the brains of rats (Sahin and Gumuslu, 2004). None of these studies reported on the amount of selenium used in their experimental designs, however, and the expression of GPX1 is highly dependent on selenium availability (Handy and Loscalzo, 2022). Indeed, we found that high maternal selenium consumption independently raised GPX1 protein content in the hippocampus of both sexes and in the female prefrontal cortex. Moreover, GPX1 increased severalfold in response to CORT in the hippocampus, and to a significantly higher level in high selenium compared to moderate selenium subjects. This demonstrates that the CORT-induced GPX1 increases are likely limited by the amount of selenium available.

Maternal CORT exposure also elevated GPX4 in all three brain regions, albeit not on the same order of magnitude as for GPX1. Highly expressed in the brain (Sasuclark et al., 2019), GPX4 is essential for neuronal health (Ursini et al., 2022) and primarily targets lipid hydroperoxides for reduction. Constitutive knockout of the GPX4 gene in mice is embryonically lethal (Imai et al., 2003; Yant et al., 2003) and the induction of brain-specific deletion causes neuronal death and severe neurological deficits (Seiler et al., 2008). GPX4 has gained increasing attention in recent years for its major role in preventing ferroptotic cell death (Stockwell, 2022). Several recent papers have reported on the ability of the synthetic glucocorticoid dexamethasone to promote ferroptosis in various models (Yang et al., 2021; Miao et al., 2022; Sun et al., 2022; Von Massenhausen et al., 2022). Thus, a generation of pro-ferroptotic conditions on the part of maternal CORT consumption may explain the increase in brain GPX4 levels observed in our study. This potential scenario is supported by a study demonstrating that ferroptotic stimuli induce a transcriptional response in neurons that involves the upregulation of several selenoproteins, including GPX4 and GPX1 (Alim et al., 2019). Taken together with the concurrent increase in GPX1, these results further suggest the possibility that administration of exogenous CORT to lactating dams can create a pro-oxidative environment in GC-responsive brain regions of the offspring.

We also observed that maternal CORT consumption increased SELENOW protein, but this effect was only seen in the hypothalamus. SELENOW is abundantly expressed in the brain of both rodents and humans (Whanger, 2009; Raman et al., 2013; Sasuclark et al., 2019). Although it does contain a thioredoxin-like motif (Zhang et al., 2019), a specific mechanism of action has yet to be uncovered, and very little is known about its function in the brain. Even less is known regarding the potential role of SELENOW in the hypothalamus. However, one study on goldfish did note that *selenow* mRNA levels decreased by food deprivation and were restored by refeeding (Chen et al., 2015). A prominent role for selenium and selenoproteins in hypothalamic function has begun to come to light, particularly in regards to the regulation of energy homeostasis (Gong et al., 2018). The fact that maternal CORT exposure increased SELENOW in the hypothalamus of offspring, but not in the other regions, suggests that the underlying mechanism differs from that for the increases in GPX1 and GPX4. Although each brain region was analyzed separately, we did not notice a stronger expression level of SELENOW in the hypothalamus compared to the hippocampus and prefrontal cortex. It is worth noting that the high selenium diet increased SELENOW levels in the prefrontal cortex of female, but not male, offspring.

There are some important factors to keep in mind regarding the experimental design of our study. The consumption of GCs by nursing dams *via* the drinking water has been demonstrated to cause the delivery of excess GCs to the offspring *via* the lactating dam’s milk (Angelucci et al., 1985). One major benefit of this is that CORT levels can be elevated in the offspring without physically disturbing the animals, thereby isolating the effects of GC activity (Catalani et al., 1993). Although we were unable to confirm the elevation of CORT levels in the bloodstream of offspring in our study due to technical limitations, the reduction in GCR levels we observed in the brains of the offspring is consistent with excessive GC exposure. Still, the potential contribution of other factors, such as the influence of maternal behavior changes caused by CORT consumption, cannot be discounted. A weakness of delivering GCs through drinking water is the inability to control the total amount of GCs being delivered as it depends on the overall water consumption of each individual dam. Oral administration of CORT in rodents is often used to model a common GC prescription regimen (Wray et al., 2019), and avoids the additional stress caused by daily injections and surgical implants. Importantly, cortisol levels in maternal plasma and breast milk are closely correlated in humans (Patacchioli et al., 1992), and breast milk GCs have been shown to influence behavioral and metabolic programming in human and rodent offspring (Hollanders et al., 2017). It must also be noted that since entire litters received one experimental treatment, the potential for inter-litter variability to have affected out results cannot be ruled out.

Our experimental design targeted PND 1–15, a time period during which the brain growth spurt (BGS) occurs in mice. This key stage of neurodevelopment that involves intense levels of cell proliferation and differentiation, synaptogenesis, and myelination of key structures (Semple et al., 2013; Chen et al., 2017) begins during the third trimester in humans (Dobbing and Sands, 1979), and researchers, therefore, often target this postnatal age range in mice to model the exposure to harmful stimuli during the BGS in humans (Workman et al., 2013). Thus, our data could have some relevance to the neurodevelopmental effects of maternal GC consumption during the later third trimester in humans. Synthetic GCs are commonly prescribed for their anti-inflammatory properties, including during pregnancy which is known to increase the risk for a variety of adverse developmental effects (Bandoli et al., 2017). Moreover, increased oxygen demand during pregnancy increases ROS production (Mihailovic et al., 2000; Pieczynska and Grajeta, 2015). Finally, the model of exposing neonates to excess GCs through maternal milk has been used in studies on the neurological impact of early life stress (ELS) events (Catalani et al., 2011). To our knowledge, there has been no previous report of a direct relationship between selenium status and the long-term neurological effects of ELS. Future studies will, therefore, include the longitudinal analysis of the behavioral outcomes of the offspring of CORT-exposed dams fed varying levels of dietary selenium. It is important to remember, however, that although there are some similarities in the progression of neurodevelopmental events between rodents and humans, they are not totally congruent and further investigation is needed to dissect the molecular processes involved.

Our results indicate that excess GC exposure can alter the expression of antioxidant selenoproteins in the developing brain. There are a very limited number of previous reports discussing the ability of GCs to regulate selenoproteins. One study conducted with human HEK-293 cells, revealed that activation of the GCR can prevent the transactivation of the *Selenop* gene (Rock and Moos, 2009). A separate study using various human lung cancer cell lines found that GCs can increase the gene expression of Gpx3, and gene analysis techniques identified a pair of glucocorticoid response elements (GREs) in the downstream regulatory region (An et al., 2016). We did not measure GPX3 levels in our study, however, since it is mainly present in the bloodstream as a secreted enzyme and is expressed in very low levels in the brain (Sasuclark et al., 2019). Degradation of the ER-resident Selenoprotein S was shown to be elevated in response to dexamethasone in human 3 T3-L1 cells undergoing differentiation into adipocytes (Kim and Kim, 2013). Finally, a recent study was conducted by Wray et al. in which adult mice were administered CORT *via* drinking water for 4 weeks (Wray et al., 2019). RNAseq analysis of the arcuate nucleus of the hypothalamus revealed an upregulation of SELENOP and the thyroid hormone-regulating selenoprotein deiodinase 2 (DIO2), as well as a downregulation of the selenium metabolism enzyme selenocysteine lyase. Considering that SELENOP serves to distribute selenium throughout the body, including to the brain (Schomburg, 2022), this could suggest an increase in brain selenium content, and is an interesting parallel to our data showing increases in GPX1, GPX4, and SELENOW in the offspring of CORT-consuming dams. Could chronic elevation of GCs induce oxidative stress in the brain that results in the body redistributing selenium towards the brain? Such a phenomenon would have particularly important implications for both pregnant and lactating individuals as they are more prone to selenium deficiency (Varsi et al., 2017; Schomburg, 2021). Further work with animal models will help answer these questions and help guide studies in humans. In conclusion, we have confirmed that early-life exposure to exogenous GCs can alter selenoprotein levels in the brain, warranting further elucidation of the underlying mechanisms.

## Data availability statement

The original contributions presented in the study are included in the article, further inquiries can be directed to the corresponding author.

## Ethics statement

The animal study was reviewed and approved by University of Hawaii at Manoa Institutional Animal Care and Use Committee.

## Author contributions

PT: investigation, formal analysis, data interpretation, data curation, writing–original draft, review and editing, and visualization. LS: data interpretation, validation, writing–review and editing, and funding acquisition. MB: data interpretation, validation, writing–review and editing, and funding acquisition. DT: conceptualization, methodology, data interpretation, data curation, validation, writing–review and editing, visualization, supervision, funding acquisition, and project administration. All authors contributed to the article and approved the submitted version.

## Funding

This work was supported by the following NIH grants: R01DK047320 (MB), F32DK124963 (DT), R01DK128390 (LS), and P20GM139753 (MB).

## Conflict of interest

The authors declare that the research was conducted in the absence of any commercial or financial relationships that could be construed as a potential conflict of interest.

## Publisher’s note

All claims expressed in this article are solely those of the authors and do not necessarily represent those of their affiliated organizations, or those of the publisher, the editors and the reviewers. Any product that may be evaluated in this article, or claim that may be made by its manufacturer, is not guaranteed or endorsed by the publisher.

## References

[ref1] Alim I. Caulfield J. T. Chen Y. Swarup V. Geschwind D. H. Ivanova E.. (2019). Selenium drives a transcriptional adaptive program to block Ferroptosis and treat stroke. Cells 177:E25. doi: 10.1016/j.cell.2019.03.032 31056284

[ref2] Ambroziak U. Hybsier S. Shahnazaryan U. Krasnodebska-Kiljanska M. Rijntjes E. Bartoszewicz Z.. (2017). Severe selenium deficits in pregnant women irrespective of autoimmune thyroid disease in An area with marginal selenium intake. J. Trace Elem. Med. Biol. 44, 186–191. doi: 10.1016/j.jtemb.2017.08.005, PMID: 28965575

[ref3] An B. C. Jung N. K. Park C. Y. Oh I. J. Choi Y. D. Park J. I.. (2016). Epigenetic and glucocorticoid receptor-mediated regulation of glutathione peroxidase 3 in lung cancer cells. Mol. Cells 39, 631–638. doi: 10.14348/molcells.2016.0164, PMID: 27484907 PMC4990756

[ref4] Angelucci L. Patacchioli F. R. Scaccianoce S. Di Sciullo A. Cardillo A. Maccari S. (1985). A model for later-life effects of perinatal drug exposure: maternal hormone mediation. Neurobehav. Toxicol. Teratol. 7, 511–517. 4080068

[ref5] Angoa-Perez M. Zagorac B. Francescutti D. M. Theis K. R. Kuhn D. M. (2021). Responses to chronic corticosterone on brain glucocorticoid receptors, adrenal gland, and gut microbiota in mice lacking neuronal serotonin. Brain Res. 1751:147190. doi: 10.1016/j.brainres.2020.147190, PMID: 33152342 PMC8650149

[ref6] Arner E. S. (2010). Selenoproteins-what unique properties can Arise with Selenocysteine in place of cysteine? Exp. Cell Res. 316, 1296–1303. doi: 10.1016/j.yexcr.2010.02.032, PMID: 20206159

[ref7] Asayama K. Hayashibe H. Dobashi K. Uchida N. Kato K. (1992). Effect of dexamethasone on antioxidant enzymes in fetal rat lungs and kidneys. Biol. Neonate 62, 136–144. doi: 10.1159/000243866, PMID: 1420613

[ref8] Bandoli G. Palmsten K. Forbess Smith C. J. Chambers C. D. (2017). A review of systemic corticosteroid use in pregnancy and the risk of select pregnancy and birth outcomes. Rheum. Dis. Clin. N. Am. 43, 489–502. doi: 10.1016/j.rdc.2017.04.013, PMID: 28711148 PMC5604866

[ref9] Behl C. Lezoualc'h F. Trapp T. Widmann M. Skutella T. Holsboer F. (1997). Glucocorticoids enhance oxidative stress-induced cell death in hippocampal neurons in vitro. Endocrinology 138, 101–106. doi: 10.1210/endo.138.1.4835, PMID: 8977391

[ref10] Cadenas E. Davies K. J. (2000). Mitochondrial free radical generation, oxidative stress, and aging. Free Radic. Biol. Med. 29, 222–230. doi: 10.1016/S0891-5849(00)00317-8, PMID: 11035250

[ref11] Casaril A. M. Domingues M. Bampi S. R. De Andrade Lourenco D. Padilha N. B. Lenardao E. J.. (2019). The selenium-containing compound 3-((4-Chlorophenyl)Selanyl)-1-methyl-1h-indole reverses depressive-like behavior induced by acute restraint stress in mice: modulation of Oxido-Nitrosative stress and inflammatory pathway. Psychopharmacology 236, 2867–2880. doi: 10.1007/s00213-018-5151-x, PMID: 30610349

[ref12] Casaril A. M. Ignasiak M. T. Chuang C. Y. Vieira B. Padilha N. B. Carroll L.. (2017). Selenium-containing Indolyl compounds: kinetics of reaction with inflammation-associated oxidants and protective effect against oxidation of extracellular matrix proteins. Free Radic. Biol. Med. 113, 395–405. doi: 10.1016/j.freeradbiomed.2017.10.344, PMID: 29055824

[ref13] Catalani A. Alema G. S. Cinque C. Zuena A. R. Casolini P. (2011). Maternal corticosterone effects on hypothalamus-pituitary-adrenal Axis regulation and behavior of the offspring in rodents. Neurosci. Biobehav. Rev. 35, 1502–1517. doi: 10.1016/j.neubiorev.2010.10.017, PMID: 21056056

[ref14] Catalani A. Marinelli M. Scaccianoce S. Nicolai R. Muscolo L. A. Porcu A.. (1993). Progeny of mothers drinking corticosterone during lactation has lower stress-induced corticosterone secretion and better cognitive performance. Brain Res. 624, 209–215. doi: 10.1016/0006-8993(93)90079-3, PMID: 8252393

[ref15] Chen V. S. Morrison J. P. Southwell M. F. Foley J. F. Bolon B. Elmore S. A. (2017). Histology atlas of the developing prenatal and postnatal mouse central nervous system, with emphasis on prenatal days E7.5 to E18.5. Toxicol. Pathol. 45, 705–744. doi: 10.1177/0192623317728134, PMID: 28891434 PMC5754028

[ref16] Chen W. Zhang Z. Dong H. Jiang X. (2015). Molecular cloning and sequence analysis of Selenoprotein W gene and its Mrna expression patterns in response to metabolic status and cadmium exposure in goldfish, Carassius Auratus. Comp. Biochem. Physiol. B Biochem. Mol. Biol. 184, 1–9. doi: 10.1016/j.cbpb.2015.01.005, PMID: 25659929

[ref17] Conrad C. D. (2008). Chronic stress-induced hippocampal vulnerability: the glucocorticoid vulnerability hypothesis. Rev. Neurosci. 19, 395–411. doi: 10.1515/revneuro.2008.19.6.395 19317179 PMC2746750

[ref18] Costantini D. Marasco V. Moller A. P. (2011). A meta-analysis of glucocorticoids as modulators of oxidative stress in vertebrates. J. Comp. Physiol. B 181, 447–456. doi: 10.1007/s00360-011-0566-2, PMID: 21416253

[ref19] Dobbing J. Sands J. (1979). Comparative aspects of the brain growth spurt. Early Hum. Dev. 3, 79–83. doi: 10.1016/0378-3782(79)90022-7, PMID: 118862

[ref20] Dougall W. C. Nick H. S. (1991). Manganese superoxide dismutase: a hepatic acute phase protein regulated by Interleukin-6 and glucocorticoids. Endocrinology 129, 2376–2384. doi: 10.1210/endo-129-5-2376, PMID: 1718727

[ref21] Du J. Wang Y. Hunter R. Wei Y. Blumenthal R. Falke C.. (2009). Dynamic regulation of mitochondrial function by glucocorticoids. Proc. Natl. Acad. Sci. U. S. A. 106, 3543–3548. doi: 10.1073/pnas.0812671106, PMID: 19202080 PMC2637276

[ref22] Godoy L. D. Rossignoli M. T. Delfino-Pereira P. Garcia-Cairasco N. De Lima Umeoka E. H. (2018). A comprehensive overview on stress neurobiology: basic concepts and clinical implications. Front. Behav. Neurosci. 12:127. doi: 10.3389/fnbeh.2018.00127, PMID: 30034327 PMC6043787

[ref23] Gong T. Torres D. J. Berry M. J. Pitts M. W. (2018). Hypothalamic redox balance and leptin signaling - emerging role of Selenoproteins. Free Radic. Biol. Med. 127, 172–181. doi: 10.1016/j.freeradbiomed.2018.02.038, PMID: 29518483 PMC6123311

[ref24] Grilo L. F. Tocantins C. Diniz M. S. Gomes R. M. Oliveira P. J. Matafome P.. (2021). Metabolic disease programming: from mitochondria to epigenetics, glucocorticoid Signalling and beyond. Eur. J. Clin. Investig. 51:E13625. doi: 10.1111/eci.13625 34060076

[ref25] Handy D. E. Loscalzo J. (2022). The role of glutathione Peroxidase-1 in health and disease. Free Radic. Biol. Med. 188, 146–161. doi: 10.1016/j.freeradbiomed.2022.06.004, PMID: 35691509 PMC9586416

[ref26] Herman J. P. Mcklveen J. M. Ghosal S. Kopp B. Wulsin A. Makinson R.. (2016). Regulation of the hypothalamic-pituitary-adrenocortical stress response. Compr. Physiol. 6, 603–621. doi: 10.1002/cphy.c150015, PMID: 27065163 PMC4867107

[ref27] Hill K. E. Motley A. K. Winfrey V. P. Burk R. F. (2014). Selenoprotein P is the major selenium transport protein in mouse Milk. PLoS One 9:E103486. doi: 10.1371/journal.pone.0103486, PMID: 25068390 PMC4113432

[ref28] Hollanders J. J. Heijboer A. C. Van Der Voorn B. Rotteveel J. Finken M. J. J. (2017). Nutritional programming by glucocorticoids in breast Milk: targets, mechanisms and possible implications. Best Pract. Res. Clin. Endocrinol. Metab. 31, 397–408. doi: 10.1016/j.beem.2017.10.001, PMID: 29221568

[ref29] Imai H. Hirao F. Sakamoto T. Sekine K. Mizukura Y. Saito M.. (2003). Early embryonic lethality caused by targeted disruption of the mouse Phgpx gene. Biochem. Biophys. Res. Commun. 305, 278–286. doi: 10.1016/S0006-291X(03)00734-4, PMID: 12745070

[ref30] Kim C. Y. Kim K. H. (2013). Dexamethasone-induced Selenoprotein S degradation is required for Adipogenesis. J. Lipid Res. 54, 2069–2082. doi: 10.1194/jlr.M034603, PMID: 23687306 PMC3708358

[ref31] Kratschmar D. V. Calabrese D. Walsh J. Lister A. Birk J. Appenzeller-Herzog C.. (2012). Suppression of the Nrf2-dependent antioxidant response by glucocorticoids and 11beta-Hsd1-mediated glucocorticoid activation in hepatic cells. PLoS One 7:E36774. doi: 10.1371/journal.pone.0036774, PMID: 22606287 PMC3350474

[ref32] Lupien S. J. Mcewen B. S. Gunnar M. R. Heim C. (2009). Effects of stress throughout the lifespan on the brain, behaviour and cognition. Nat. Rev. Neurosci. 10, 434–445. doi: 10.1038/nrn2639 19401723

[ref33] Mcewen B. S. (2007). Physiology and neurobiology of stress and adaptation: central role of the brain. Physiol. Rev. 87, 873–904. doi: 10.1152/physrev.00041.2006, PMID: 17615391

[ref34] Mcewen B. S. Weiss J. M. Schwartz L. S. (1968). Selective retention of corticosterone by limbic structures in rat brain. Nature 220, 911–912. doi: 10.1038/220911a0, PMID: 4301849

[ref35] Mcintosh L. J. Cortopassi K. M. Sapolsky R. M. (1998a). Glucocorticoids may Alter antioxidant enzyme capacity in the brain: Kainic acid studies. Brain Res. 791, 215–222. doi: 10.1016/S0006-8993(98)00104-8, PMID: 9593900

[ref36] Mcintosh L. J. Hong K. E. Sapolsky R. M. (1998b). Glucocorticoids may Alter antioxidant enzyme capacity in the brain: baseline studies. Brain Res. 791, 209–214. doi: 10.1016/S0006-8993(98)00115-2, PMID: 9593898

[ref37] Mcintosh L. J. Sapolsky R. M. (1996). Glucocorticoids increase the accumulation of reactive oxygen species and enhance Adriamycin-induced toxicity in neuronal culture. Exp. Neurol. 141, 201–206. doi: 10.1006/exnr.1996.0154, PMID: 8812153

[ref38] Miao W. He L. Zhang Y. Zhu X. Jiang Y. Liu P.. (2022). Ferroptosis is partially responsible for dexamethasone-induced T cell ablation, but not osteoporosis in larval zebrafish. Ecotoxicol. Environ. Saf. 242:113872. doi: 10.1016/j.ecoenv.2022.113872, PMID: 35835076

[ref39] Mihailovic M. Cvetkovic M. Ljubic A. Kosanovic M. Nedeljkovic S. Jovanovic I.. (2000). Selenium and malondialdehyde content and glutathione peroxidase activity in maternal and umbilical cord blood and amniotic fluid. Biol. Trace Elem. Res. 73, 47–54. doi: 10.1385/BTER:73:1:47, PMID: 10949968

[ref40] Monsen E. R. (2000). Dietary reference intakes for the antioxidant nutrients: vitamin C, vitamin E, selenium, and carotenoids. J. Am. Diet. Assoc. 100, 637–640. doi: 10.1016/S0002-8223(00)00189-9 10863565

[ref41] Morella I. M. Brambilla R. More L. (2022). Emerging roles of brain metabolism in cognitive impairment and neuropsychiatric disorders. Neurosci. Biobehav. Rev. 142:104892. doi: 10.1016/j.neubiorev.2022.104892, PMID: 36181925

[ref42] Nicholson J. L. Toh P. Alfulaij N. Berry M. J. Torres D. J. (2022). New insights on Selenoproteins and neuronal function. Free Radic. Biol. Med. 190, 55–61. doi: 10.1016/j.freeradbiomed.2022.07.021, PMID: 35948259

[ref43] Okamoto K. Tanaka H. Makino Y. Makino I. (1998). Restoration of the glucocorticoid receptor function by the phosphodiester compound of vitamins C and E, Epc-K1 (L-ascorbic acid 2-[3,4-Dihydro-2,5,7,8-Tetramethyl-2-(4,8,12-Trimethyltridecyl)-2h-1-Benzopyran-6 -Yl hydrogen phosphate] potassium salt), via a redox-dependent mechanism. Biochem. Pharmacol. 56, 79–86. doi: 10.1016/S0006-2952(98)00121-X, PMID: 9698091

[ref44] Okamoto K. Tanaka H. Ogawa H. Makino Y. Eguchi H. Hayashi S.. (1999). Redox-dependent regulation of nuclear import of the glucocorticoid receptor. J. Biol. Chem. 274, 10363–10371. doi: 10.1074/jbc.274.15.10363, PMID: 10187825

[ref45] Patacchioli F. R. Cigliana G. Cilumbriello A. Perrone G. Capri O. Alema G. S.. (1992). Maternal plasma and Milk free cortisol during the first 3 days of breast-feeding following spontaneous delivery or elective cesarean section. Gynecol. Obstet. Investig. 34, 159–163. doi: 10.1159/000292751 1427417

[ref46] Patel R. Mcintosh L. Mclaughlin J. Brooke S. Nimon V. Sapolsky R. (2002). Disruptive effects of glucocorticoids on glutathione peroxidase biochemistry in hippocampal cultures. J. Neurochem. 82, 118–125. doi: 10.1046/j.1471-4159.2002.00948.x, PMID: 12091472

[ref47] Patterson S. Zee J. Wiseman S. Hecker M. (2017). Effects of chronic exposure to dietary Selenomethionine on the physiological stress response in juvenile White sturgeon (Acipenser Transmontanus). Aquat. Toxicol. 186, 77–86. doi: 10.1016/j.aquatox.2017.02.003, PMID: 28260669

[ref48] Pieczynska J. Grajeta H. (2015). The role of selenium in human conception and pregnancy. J. Trace Elem. Med. Biol. 29, 31–38. doi: 10.1016/j.jtemb.2014.07.003, PMID: 25175508

[ref49] Raman A. V. Pitts M. W. Seyedali A. Hashimoto A. C. Bellinger F. P. Berry M. J. (2013). Selenoprotein W expression and regulation in mouse brain and neurons. Brain Behav. 3, 562–574. doi: 10.1002/brb3.159, PMID: 24392277 PMC3869984

[ref50] Rock C. Moos P. J. (2009). Selenoprotein P regulation by the glucocorticoid receptor. Biometals 22, 995–1009. doi: 10.1007/s10534-009-9251-2, PMID: 19513589 PMC3039700

[ref51] Sahin E. Gumuslu S. (2004). Alterations in brain antioxidant status, protein oxidation and lipid peroxidation in response to different stress models. Behav. Brain Res. 155, 241–248. doi: 10.1016/j.bbr.2004.04.022, PMID: 15364483

[ref52] Sapolsky R. M. (1986). Glucocorticoid toxicity in the hippocampus: reversal by supplementation with brain fuels. J. Neurosci. 6, 2240–2244. doi: 10.1523/JNEUROSCI.06-08-02240.1986, PMID: 3746406 PMC6568753

[ref53] Sapolsky R. M. Romero L. M. Munck A. U. (2000). How do glucocorticoids influence stress responses? Integrating permissive, suppressive, stimulatory, and preparative actions. Endocr. Rev. 21, 55–89. doi: 10.1210/edrv.21.1.0389 10696570

[ref54] Sasuclark A. R. Khadka V. S. Pitts M. W. (2019). Cell-type specific analysis of selenium-related genes in brain. Antioxidants (Basel) 8:120. doi: 10.3390/antiox8050120 31060314 PMC6562762

[ref55] Sato H. Takahashi T. Sumitani K. Takatsu H. Urano S. (2010). Glucocorticoid generates Ros to induce oxidative injury in the hippocampus, leading to impairment of cognitive function of rats. J. Clin. Biochem. Nutr. 47, 224–232. doi: 10.3164/jcbn.10-58, PMID: 21103031 PMC2966932

[ref56] Schomburg L. (2021). Selenium deficiency due to diet, pregnancy, severe illness, or Covid-19-a preventable trigger for autoimmune disease. Int. J. Mol. Sci. 22:8532. doi: 10.3390/ijms22168532, PMID: 34445238 PMC8395178

[ref57] Schomburg L. (2022). Selenoprotein P–selenium transport protein, enzyme and biomarker of selenium status. Free Radic Biol Med 191, 150–163. doi: 10.1016/j.freeradbiomed.2022.08.022 36067902

[ref58] Schweizer U. Fabiano M. (2022). Selenoproteins in brain development and function. Free Radic. Biol. Med. 190, 105–115. doi: 10.1016/j.freeradbiomed.2022.07.022, PMID: 35961466

[ref59] Seiler A. Schneider M. Forster H. Roth S. Wirth E. K. Culmsee C.. (2008). Glutathione peroxidase 4 senses and translates oxidative stress into 12/15-lipoxygenase dependent- and Aif-mediated cell death. Cell Metab. 8, 237–248. doi: 10.1016/j.cmet.2008.07.005, PMID: 18762024

[ref60] Semple B. D. Blomgren K. Gimlin K. Ferriero D. M. Noble-Haeusslein L. J. (2013). Brain development in rodents and humans: identifying benchmarks of maturation and vulnerability to injury across species. Prog. Neurobiol. 106-107, 1–16. doi: 10.1016/j.pneurobio.2013.04.001, PMID: 23583307 PMC3737272

[ref61] Shimba A. Ikuta K. (2020). Control of immunity by glucocorticoids in health and disease. Semin. Immunopathol. 42, 669–680. doi: 10.1007/s00281-020-00827-8, PMID: 33219395

[ref62] Solovyev N. D. (2015). Importance of selenium and Selenoprotein for brain function: from antioxidant protection to neuronal Signalling. J. Inorg. Biochem. 153, 1–12. doi: 10.1016/j.jinorgbio.2015.09.003, PMID: 26398431

[ref63] Spiers J. G. Chen H. J. Sernia C. Lavidis N. A. (2014). Activation of the hypothalamic-pituitary-adrenal stress Axis induces cellular oxidative stress. Front. Neurosci. 8:456. doi: 10.3389/fnins.2014.00456 25646076 PMC4298223

[ref64] Stein-Behrens B. A. Lin W. J. Sapolsky R. M. (1994). Physiological elevations of glucocorticoids potentiate glutamate accumulation in the hippocampus. J. Neurochem. 63, 596–602. 7913489 10.1046/j.1471-4159.1994.63020596.x

[ref65] Stockwell B. R. (2022). Ferroptosis turns 10: emerging mechanisms, physiological functions, and therapeutic applications. Cells 185, 2401–2421. doi: 10.1016/j.cell.2022.06.003, PMID: 35803244 PMC9273022

[ref66] Sun F. Zhou J. L. Liu Z. L. Jiang Z. W. Peng H. (2022). Dexamethasone induces Ferroptosis via P53/Slc7a11/Gpx4 pathway in glucocorticoid-induced osteonecrosis of the femoral head. Biochem. Biophys. Res. Commun. 602, 149–155. doi: 10.1016/j.bbrc.2022.02.112, PMID: 35276555

[ref67] Tanaka H. Makino Y. Okamoto K. Iida T. Yan K. Yoshikawa N. (1999). Redox regulation of the glucocorticoid receptor. Antioxid. Redox Signal. 1, 403–423. doi: 10.1089/ars.1999.1.4-403 11233142

[ref68] Tashima Y. Terui M. Itoh H. Mizunuma H. Kobayashi R. Marumo F. (1989). Effect of selenite on glucocorticoid receptor. J. Biochem. 105, 358–361. doi: 10.1093/oxfordjournals.jbchem.a122668, PMID: 2732210

[ref69] Torres D. J. Alfulaij N. Berry M. J. (2021). Stress and the brain: An emerging role for selenium. Front. Neurosci. 15:666601. doi: 10.3389/fnins.2021.666601, PMID: 33935643 PMC8081839

[ref70] Treccani G. Musazzi L. Perego C. Milanese M. Nava N. Bonifacino T.. (2014). Stress and corticosterone increase the readily releasable Pool of glutamate vesicles in synaptic terminals of prefrontal and frontal cortex. Mol. Psychiatry 19, 433–443. doi: 10.1038/mp.2014.5, PMID: 24535456

[ref71] Tsuji P. A. Santesmasses D. Lee B. J. Gladyshev V. N. Hatfield D. L. (2021). Historical roles of selenium and Selenoproteins in health and development: the good, the bad and the ugly. Int. J. Mol. Sci. 23:5. doi: 10.3390/ijms23010005, PMID: 35008430 PMC8744743

[ref72] Ursini F. Bosello Travain V. Cozza G. Miotto G. Roveri A. Toppo S.. (2022). A White paper on phospholipid Hydroperoxide glutathione peroxidase (Gpx4) forty years later. Free Radic. Biol. Med. 188, 117–133. doi: 10.1016/j.freeradbiomed.2022.06.227, PMID: 35718302

[ref73] Varsi K. Bolann B. Torsvik I. Rosvold Eik T. C. Hol P. J. Bjorke-Monsen A. L. (2017). Impact of maternal selenium status on infant outcome during the first 6 months of life. Nutrients 9:486. doi: 10.3390/nu9050486, PMID: 28492511 PMC5452216

[ref74] Von Massenhausen A. Zamora Gonzalez N. Maremonti F. Belavgeni A. Tonnus W. Meyer C.. (2022). Dexamethasone sensitizes to Ferroptosis by glucocorticoid receptor-induced Dipeptidase-1 expression and glutathione depletion. Sci. Adv. 8:Eabl8920. doi: 10.1126/sciadv.abl8920, PMID: 35108055 PMC8809683

[ref75] Whanger P. D. (2009). Selenoprotein expression and function-Selenoprotein W. Biochim. Biophys. Acta 1790, 1448–1452. doi: 10.1016/j.bbagen.2009.05.010, PMID: 19464347

[ref76] Workman A. D. Charvet C. J. Clancy B. Darlington R. B. Finlay B. L. (2013). Modeling transformations of neurodevelopmental sequences across mammalian species. J. Neurosci. 33, 7368–7383. doi: 10.1523/JNEUROSCI.5746-12.2013, PMID: 23616543 PMC3928428

[ref77] Wray J. R. Davies A. Sefton C. Allen T. J. Adamson A. Chapman P.. (2019). Global transcriptomic analysis of the arcuate nucleus following chronic glucocorticoid treatment. Mol Metab 26, 5–17. doi: 10.1016/j.molmet.2019.05.008, PMID: 31176677 PMC6667392

[ref78] Yang R. Z. Xu W. N. Zheng H. L. Zheng X. F. Li B. Jiang L. S.. (2021). Exosomes derived from vascular endothelial cells antagonize glucocorticoid-induced osteoporosis by inhibiting Ferritinophagy with resultant limited Ferroptosis of osteoblasts. J. Cell. Physiol. 236, 6691–6705. doi: 10.1002/jcp.30331, PMID: 33590921

[ref79] Yant L. J. Ran Q. Rao L. Van Remmen H. Shibatani T. Belter J. G.. (2003). The Selenoprotein Gpx4 is essential for mouse development and protects from radiation and oxidative damage insults. Free Radic. Biol. Med. 34, 496–502. doi: 10.1016/S0891-5849(02)01360-6, PMID: 12566075

[ref80] You J. M. Yun S. J. Nam K. N. Kang C. Won R. Lee E. H. (2009). Mechanism of glucocorticoid-induced oxidative stress in rat hippocampal slice cultures. Can. J. Physiol. Pharmacol. 87, 440–447. doi: 10.1139/Y09-027, PMID: 19526038

[ref81] Zachara B. A. Wasowicz W. Gromadzinska J. Sklodowska M. Krasomski G. (1986). Glutathione peroxidase activity, selenium, and lipid peroxide concentrations in blood from a healthy polish population: I. maternal and cord blood. Biol. Trace Elem. Res. 10, 175–187. doi: 10.1007/BF02795616, PMID: 24254392

[ref82] Zhang L. Zhu J. H. Zhang X. Cheng W. H. (2019). The Thioredoxin-like family of Selenoproteins: implications in aging and age-related degeneration. Biol. Trace Elem. Res. 188, 189–195. doi: 10.1007/s12011-018-1521-9, PMID: 30229511

